# Augmenting Venetoclax Activity Through Signal Transduction in AML

**DOI:** 10.33696/signaling.4.085

**Published:** 2023

**Authors:** Ian Michael Bouligny, Keri Renee Maher, Steven Grant

**Affiliations:** 1 Virginia Commonwealth University Massey Cancer Center, Division of Hematology and Oncology, Department of Internal Medicine, 1300 E. Marshall St., Richmond, VA, USA

**Keywords:** Acute myeloid leukemia, Akt pathway, Apoptotic pathways, B-cell lymphoma 2, FMS-like tyrosine kinase 3, Isocitrate dehydrogenase 1, Isocitrate dehydrogenase 2, MAPK pathway cell signaling, Signal transduction cascades

## Abstract

Venetoclax, a small-molecule B-cell lymphoma 2 (BCL-2) inhibitor, selectively eradicates leukemic stem cells (LSCs). While venetoclax has revolutionized the treatment of acute myeloid leukemia (AML), treatment failure and disease relapse are common. Mechanisms underlying venetoclax resistance are surprisingly heterogeneous. Venetoclax resistance encompasses a spectrum of genetic and epigenetic changes, with numerous pathways contributing to the upregulation of additional anti-apoptotic proteins. In this review, we address the mechanisms of venetoclax resistance in the context of signal transduction. We emphasize how aberrant cell signaling impairs apoptosis and predisposes to venetoclax failure.

Commonly activated pathways, such as FLT3, PI3K/AKT/mTOR, and RAS, contribute to upregulated anti-apoptotic mediators and are frequently responsible for refractory disease or disease relapse. We highlight novel combination strategies aimed at disabling constitutively active signal transduction to augment response and overcome venetoclax resistance.

## Introduction

Acute myeloid leukemia is a heterogeneous hematologic malignancy characterized by a maturation arrest of hematopoietic precursors [1]. Because AML is primarily a disease of older adults occurring at a median age of 68 years, many are ineligible for intensive chemotherapy [2,3]. Intensity strategies offer a survival benefit in this population. Low-dose cytarabine or hypomethylating agents, such as azacitidine or decitabine, remain a mainstay of lower-intensity treatment with a median survival of 5 – 9 months, varying by cytogenetic and molecular risk factors [4-6]. There is a persistent interest in intensifying lower-intensity treatment strategies to improve survival in the elderly population. The addition of venetoclax, a BCL-2 inhibitor, to a hypomethylating agent or low-dose cytarabine improves outcomes in patients ineligible for intensive chemotherapy, and the use of venetoclax-based regimens is now routine [5]. The race is ongoing to further characterize subsets of AML associated with improved survival when treated with venetoclax-based strategies.

Specific molecular subsets respond favorably to venetoclax augmentation. For example, AML with mutated isocitrate dehydrogenase (*IDH1* or *IDH2*) is associated with a survival benefit when treated with venetoclax and azacitidine compared to azacitidine alone (hazard ratio for death, 0.34; 95% CI, 0.20 - 0.60) [5]. In contrast, other molecular subsets — commonly those with mutations in signal transduction — are more likely to be resistant to treatment. AML with mutated FMS-like tyrosine kinase 3 (*FLT3*), rat sarcoma (*RAS*), or tyrosine-protein phosphatase non-receptor type 11 (*PTPN11*) are associated with decreased survival and higher rates of relapse following treatment with lower-intensity venetoclax-based strategies [7-11].

An emerging topic of interest is the modulation of signal transduction to circumvent venetoclax resistance. The FDA-approved FLT3 inhibitors — midostaurin and gilteritinib — are being evaluated in clinical trials in combination with venetoclax and a hypomethylating agent to reduce the incidence of relapse and improve survival [12,13]. Similarly, the use of mitogen-activated protein kinase (MAPK) pathway inhibitors, such as trametinib or cobimetinib, with venetoclax-based strategies is emerging; triplet combinations, however, are limited by toxicity [14-16]. Dose-limiting toxicities have prompted interest in developing more active, specific inhibitors of FLT3 and pathways downstream of PI3K and RAS to reduce venetoclax resistance and improve treatment tolerability. Consequently, numerous novel venetoclax-based combinations are surfacing. Following an overview of apoptosis, this review summarizes strategies to augment venetoclax response and reduce resistance, emphasizing the modulation of the commonly implicated signal transduction pathways: FLT3, PI3K, and RAS.

## Regulation and Modulation of Apoptosis

### Regulation of apoptosis

Apoptosis is a tightly regulated process of cell death, and aberrations of apoptosis are central to LSC persistence [17]. Understanding strategies that eradicate LSCs requires understanding the critical regulators of apoptosis — the BCL-2 family proteins. The BCL-2 family proteins are involved in a delicate interplay of mediators that moderate the mitochondrial (or intrinsic) apoptotic pathway. They are broadly categorized into pro-apoptotic and anti-apoptotic mediators.

Two prominent pro-apoptotic BCL-2 proteins are BAX and BAK. Activation of BAX and BAK and subsequent dimer formation results in pore-forming subunits in the mitochondria, consequently increasing the mitochondrial outer membrane permeability [18,19]. Due to increased mitochondrial permeability, egress of apoptotic effectors from the mitochondria into the cytosol occurs — with the most notable effector being cytochrome *c* [20]. Cytochrome *c* then initiates the intrinsic pathway through the activation of caspase 9 and caspase 3, culminating in the cleavage of cytosolic and nuclear proteins and the procession of cell death [21].

Additional BCL-2 family proteins potentiate apoptosis; they contain homologous structures to BAX and BAK, known as BCL-2 homology (BH) motifs. There are four BCL-2 homology motifs: BH1, BH2, BH3, and BH4 [22]. These additional subsets of BCL-2 family proteins share the BH3 homology domain and indirectly *sensitize* apoptosis (BAD, BIK, HRK, NOXA) or directly *activate* apoptosis (BIM, BID, PUMA) [23-25]. After binding these apoptotic sensitizers and activators, the anti-apoptotic proteins release bound BAX and BAK, allowing apoptosis to proceed. Additionally, BH3 apoptotic activators (BIM, BID, PUMA) directly activate BAX and BAK.

**Figure 1.**
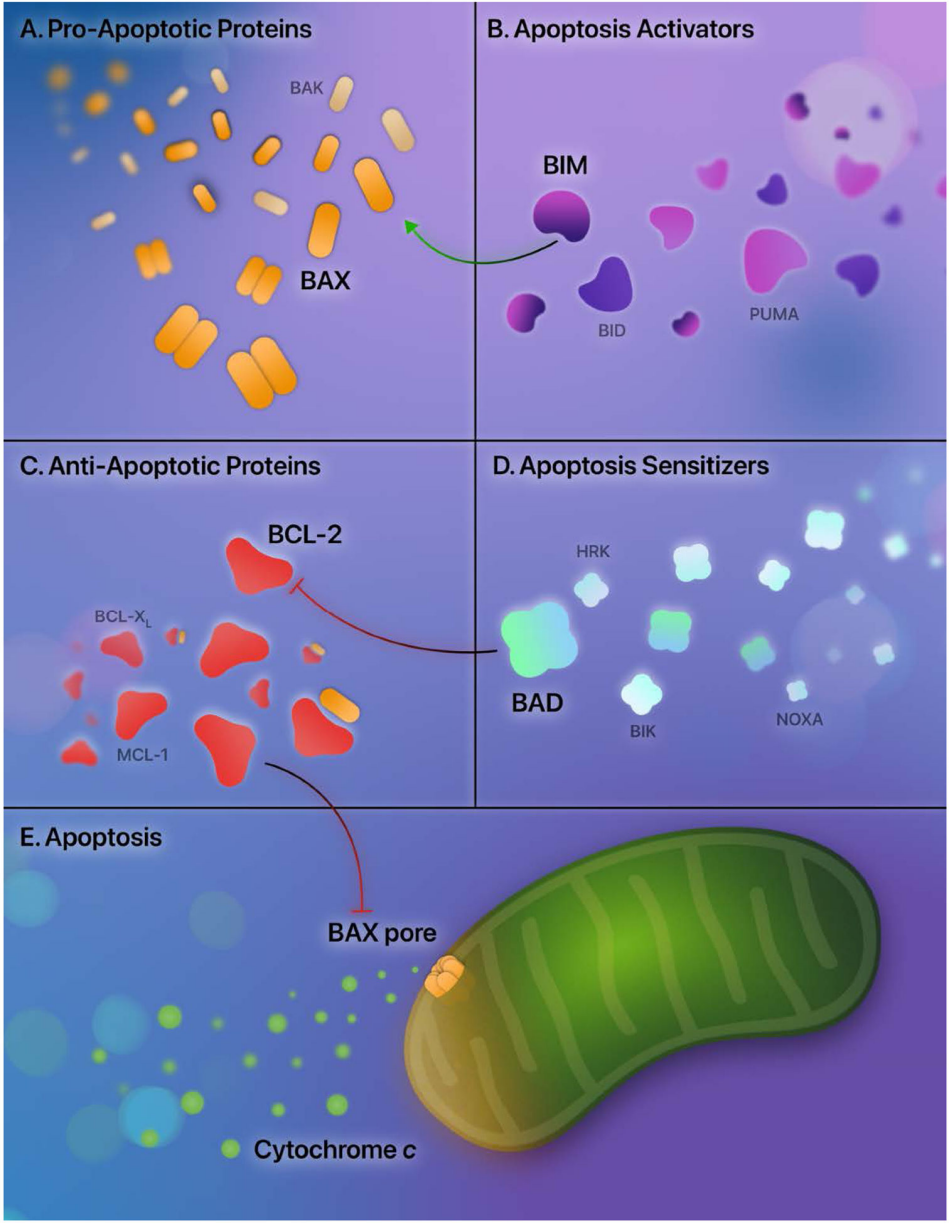
The apoptotic mediators. **A.** The primary pro-apoptotic proteins are BAX and BAK. In pro-apoptotic states, dimer formation and subsequent oligomerization occur, resulting in the initial steps of the formation of a pore subunit. **B.** The apoptosis activators are BIM, BID, and PUMA, which directly activate BAX and BAK. **C.** The anti-apoptotic proteins are BCL-2, MCL-1, BCL-XL, as well as BCL-w and BFL-1 (not pictured). These proteins bind BAX and BAK and result in the inhibition of apoptosis. **D.** The apoptotic sensitizers are BAD, BIK, HRK, and NOXA; they bind to the anti-apoptotic proteins and release bound BAX and BAK, allowing apoptosis to proceed. **E.** Apoptosis proceeds through the formation of a mitochondrial outer membrane pore, increasing mitochondrial permeability and allowing the escape of cytochrome *c*.

In contrast to the pro-apoptotic mediators, the anti-apoptotic BCL-2 proteins bind BAX and BAK, rendering them unable to drive the intrinsic pathway. The anti-apoptotic proteins include BCL-2, BCL-X_L_, BCL-w, BFL-1, and MCL-1. By binding to BAX and BAK, BCL-2 and BCL-X_L_ inhibit apoptosis and block the subsequent formation of the mitochondrial pore-forming subunits [18,26,27]. In addition, anti-apoptotic proteins sequester the BH3-only sensitizing and activating proteins, further reducing the procession of the mitochondrial apoptotic pathway [22]. An overview of these apoptotic regulators is provided in Figure 1.

### Modulation of apoptosis: BCL-2 inhibition

Promoting leukemic cell death by activating apoptosis is an attractive therapeutic strategy. Owing to the overexpression of BCL-2 in LSCs, selective inhibition of BCL-2 leads to the sequestration of BAX and BAK and induces the eradication of quiescent LSCs [17]. This discovery led to the development of the BH3 mimetics — which free the pro-apoptotic mediators by disrupting the binding of the BH3 motif to anti-apoptotic proteins, allowing apoptosis to proceed.

Navitoclax was one of several BH3 mimetics investigated shortly after the discovery of BCL-2 inhibition as a therapeutic strategy. Navitoclax selectively targets BCL-2, BCL-w, and BCL-X_L_ [22]. However, BCL-X_L_ is a platelet pro-survival protein, and inhibition of BCL-X_L_ by navitoclax resulted in dose-limiting thrombocytopenia [28]. Rates of high-grade thrombocytopenia reduced the enthusiasm for further clinical development in hematological malignancies. This led to the investigation of venetoclax in clinical trials, which binds to BCL-2, but not to BCL-X_L_. Soon afterward, the landmark VIALE-A trial demonstrated the superiority of venetoclax and 7-day azacitidine compared to azacitidine alone, and cemented the use of combination therapy in patients with AML ineligible for intensive induction [5].

There is, however, a critical caveat to the use of venetoclax. Like navitoclax, venetoclax does not inhibit the anti-apoptotic mediator MCL-1; it also does not inhibit BFL-1 or BCL-X_L_. Indeed, the upregulation of these anti-apoptotic proteins contributes directly to venetoclax resistance, and the downregulation of MCL-1 or BCL-X_L_ restores venetoclax sensitivity [29]. The upregulation of MCL-1 is common in leukemic cells following venetoclax exposure, and elevations in MCL-1 subside with venetoclax discontinuation [30]. The reduction in MCL-1 following venetoclax cessation forms the basis of a strategic molecular rationale to reduce venetoclax resistance: stopping or shortening the duration of venetoclax after a maximal response is reached.

Venetoclax resistance is remarkably heterogeneous. Elevation of anti-apoptotic mediators is a frequent accompaniment of venetoclax-resistant disease. Many paths lead to persistent cell survival following venetoclax exposure, including aberrations in epigenetics, gene amplifications, or BCL-2 family mutations [31,32] — but perhaps the path most appropriate to target is constitutively active signal transduction.

## FLT3

### Overview of FLT3

FMS-like tyrosine kinase 3 (FLT3) is among the most commonly mutated and well-understood signaling pathways in AML. FLT3 is a ligand-activated transmembrane tyrosine kinase that feeds into downstream pathways, including PI3K, RAS, and STAT5, promoting cell proliferation and survival [33]. The FLT3 receptor shares a high degree of homology with KIT, FMS, and PDGFR receptors — all of which regulate hematopoietic maturation and differentiation [34].

The two most commonly observed activating mutations in *FLT3* are an internal tandem duplication (*FLT3*-ITD) of the juxtamembrane domain and tyrosine kinase domain mutations (*FLT3*-TKD) in the activation loop, although many other *FLT3* mutations exist [35]. *FLT3*-ITD and *FLT3*-TKD lead to constitutive activation of FLT3 kinase, driving leukemic proliferation and survival. As *FLT3*^mut^ AML has an increased risk of relapse and shorter overall survival — particularly for *FLT3*-ITD [36,37] — there has been relentless interest in improving outcomes in this cohort of AML.

Sorafenib, a multi-kinase inhibitor with activity against *FLT3*-ITD but not *FLT3*-TKD [38], significantly prolonged event-free survival when combined with standard-of-care chemotherapy [39]. Subsequently, midostaurin, a first-generation multi-kinase inhibitor active against *FLT3*-ITD and *FLT3*-TKD, improved overall survival when combined with an anthracycline and cytarabine [40]. Midostaurin, however, lacks specificity for *FLT3*-ITD, which led to the development of more specific and potent next-generation FLT3 inhibitors, including quizartinib, crenolanib, and gilteritinib [41]. The most notable of these is gilteritinib, which resulted in significantly longer overall survival and higher remission rates than salvage chemotherapy in *FLT3*^mut^ AML [42].

Outcomes of *FLT3*^mut^ AML are being investigated in the context of venetoclax-based induction without a FLT3 inhibitor. A recent subgroup analysis of the VIALE-A trial analyzed 42 patients with *FLT3* mutations treated with venetoclax and azacitidine. The median overall survival of *FLT3*^mut^ AML was shorter at 12.5 months compared to 14.7 months for wild-type *FLT3* [43], suggesting some degree of *FLT3*-mediated venetoclax resistance. Additionally, new *FLT3* mutations emerge following venetoclax treatment in patients without a *FLT3* mutation at diagnosis, suggesting that *FLT3* may be responsible for resistance by an adaptive mechanism [44]. These observations provided the groundwork to refine outcomes of *FLT3*^mut^ AML treated with venetoclax-based regimens.

### Overcoming *FLT3* resistance

Venetoclax resistance in *FLT3* mutants occurs through several mechanisms in addition to the emergence of new *FLT3* mutations. As venetoclax selectively inhibits BCL-2, upregulation of anti-apoptotic proteins other than BCL-2 is a primary cause of resistance. Indeed, constitutive FLT3 signaling upregulates the anti-apoptotic mediator MCL-1 [45]; siRNA-mediated MCL-1 inhibition restores the sensitivity of *FLT3*-ITD AML to therapy [45].

**Figure 2.**
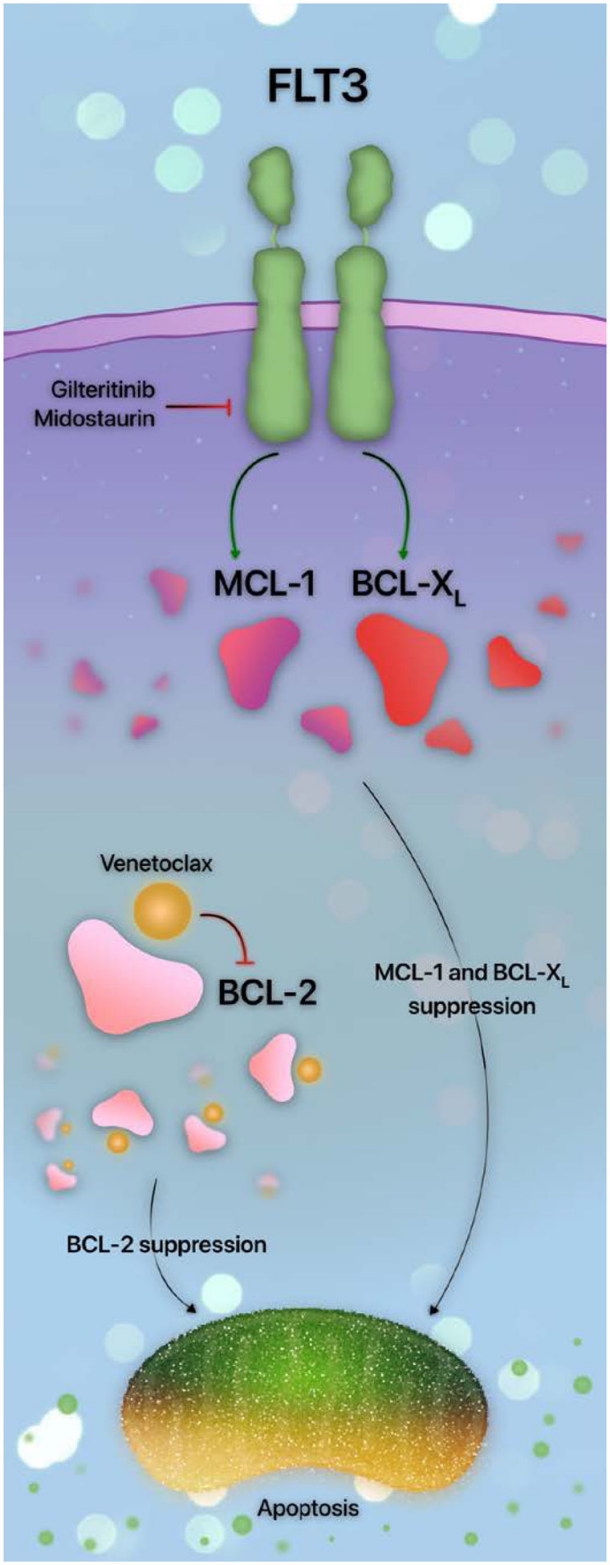
The FLT3 signaling pathway in apoptosis. Activation of FLT3 results in increased MCL-1 and BCL-X_L_, which are reversed with FLT3 inhibition with agents such as gilteritinib or midostaurin. BCL-2 inhibition with venetoclax synergizes with FLT3 inhibitor-mediated MCL-1 and BCL-X_L_ suppression, culminating in potent apoptosis.

Additional avenues of *FLT3*-mediated venetoclax resistance are similar to MCL-1 upregulation. The activity of another anti-apoptotic mediator, BCL-X_L_, is maintained in *FLT3*-ITD, resulting in leukemic stem cell persistence and survival. Targeting *FLT3*-ITD results in BCL-X_L_ reduction [46]. Together, the upregulation of MCL-1 and BCL-X_L_ appears to be an indirect consequence of downstream activation of PI3K, RAS, and STAT5 [7,46,47]. Therefore, as multiple parallel pathways are activated, the most efficacious way to overcome FLT3-mediated resistance is by targeting FLT3 rather than its downstream effectors [46]. A summary of the interplay between FLT3 and the apoptotic pathway is provided in Figure 2.

The observation that *FLT3* mutations upregulate alternative anti-apoptotic mediators led to the design of several studies investigating the efficacy of venetoclax and FLT3 inhibition. Venetoclax with sorafenib, midostaurin, or gilteritinib led to synergistic apoptosis in *FLT3*^mut^ AML through the downregulation of MCL-1 [48,49]. These observations support the hypothesis that the downregulation of pro-survival mediators other than BCL-2 may overcome venetoclax resistance. The pre-clinical demonstration of synergistic efficacy between FLT3 and BCL-2 inhibition prompted the trial design of intensifying azacitidine and venetoclax with FLT3 inhibitors.

Early phase trials of gilteritinib with 7-day azacitidine and venetoclax demonstrated an overall response rate of 100% in newly diagnosed *FLT3*^mut^ AML, with no relapses observed during the follow-up period [13]. In the relapsed or refractory setting, the overall response rate was 67%, with 44% proceeding to allogeneic stem cell transplant [13]. Prolonged myelosuppression was the dose-limiting toxicity, suggesting that strategies to refine the dose or duration of drug exposure may minimize treatment-related complications.

A similar study of sorafenib, midostaurin, or gilteritinib with 10-day decitabine and venetoclax yielded a composite complete remission rate of 92% in newly diagnosed AML, and 56% were negative for measurable residual disease (MRD). The overall survival was not reached at a median follow-up time of 14.5 months [12]. The composite complete remission rate was 62% in the relapsed or refractory setting, although the median overall survival was shorter at 6.8 months [12]. Further trials are ongoing to determine the optimal *FLT3* inhibitor, hypomethylating agent backbone, and duration of drug exposure to balance efficacy and toxicity. Nevertheless, preliminary results in the front-line setting are impressive. In relapsed or refractory AML, the combination approach is more problematic. The emergence of new *FLT3* mutations frequently hampers outcomes through clonal evolution or constitutive activation of downstream parallel signaling pathways, such as PI3K or RAS.

## Overview of PI3K

### Overview of PI3K/AKT/mTOR

Cell signaling through the phosphatidylinositol-3-kinase (PI3K)/AKT/mammalian target of rapamycin (mTOR) is one of the most central intracellular pathways in cancer. Mutations in receptor tyrosine kinases — such as FLT3 — or GTPases frequently lead to upregulation of the PI3K pathway and are associated with inferior overall survival in AML [50]. PI3K is a plasma-associated lipid kinase that converts phosphatidylinositol 4,5-bisphosphate (PIP2) to phosphatidylinositol 3,4,5-triphosphate (PIP3). PIP3 recruits lipid-binding proteins to the cell membrane and localizes AKT, activating mTOR and additional downstream pathways, culminating in cell growth, proliferation, and survival [51].

AKT-mediated cell survival occurs directly through interactions with the apoptosis-regulating proteins. AKT, a protein kinase, phosphorylates numerous substrates, including the apoptotic sensitizer BAD. Phosphorylated BAD frees BCL-2 and BCL-X_L_, allowing these anti-apoptotic proteins to block the progression of mitochondrial outer membrane permeability and subsequent cell death [52,53]. Therefore, cell survival is the net effect of PI3K/AKT signaling on apoptosis.

PI3K inhibition is unimpressive as monotherapy, and trials involving this class of agents have been limited by excessive toxicity. Gedatolisib, a dual PI3K/mTOR inhibitor, was evaluated in relapsed or refractory AML, and no objective response or clinical benefit was observed [54]. Consequently, there has been growing interest in modulating other effectors downstream from PI3K. Triciribine, an AKT activation inhibitor, was evaluated in an early phase study, demonstrating reduction of BAD and induction of cell death [55]. Clinical trials combining triciribine with chemotherapy or additional targeted agents are ongoing or planned.

**Figure 3.**
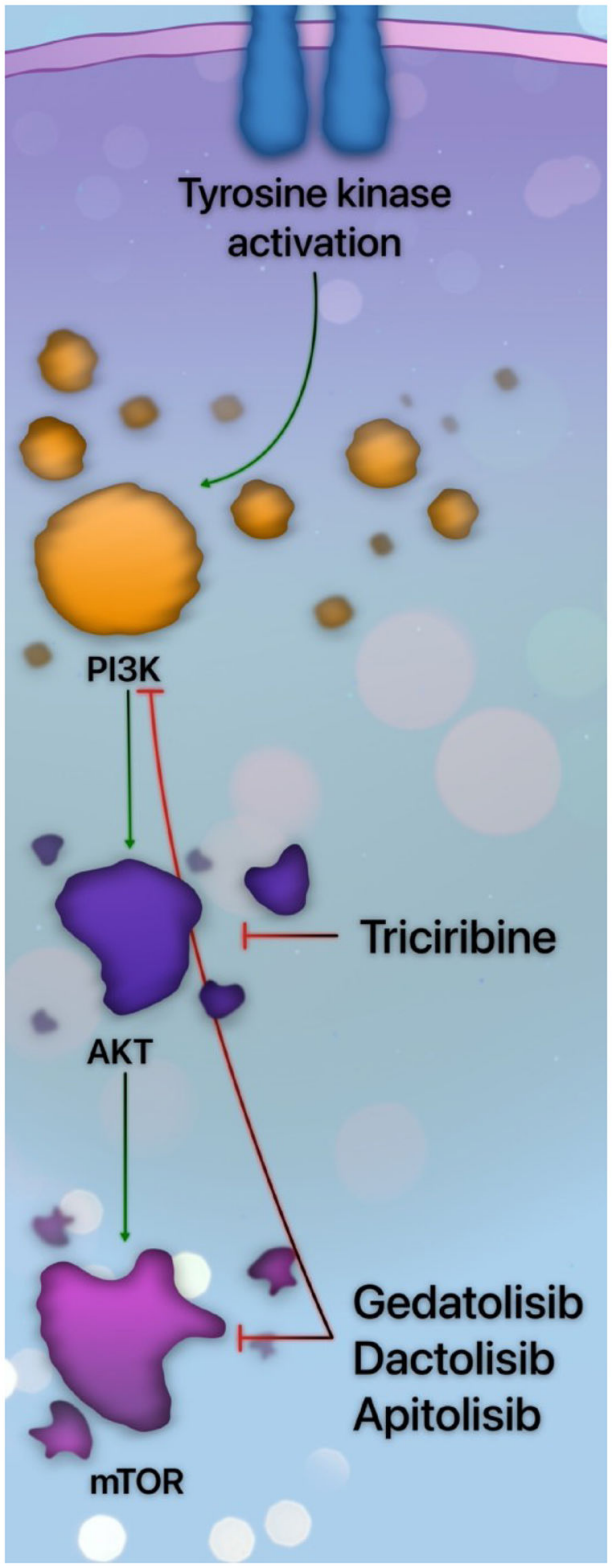
The PI3K pathway inhibitors in AML. Activation of a tyrosine kinase, such as FLT3 or KIT, leads to PI3K pathway activation. PI3K then phosphorylates AKT, which then sequentially activates mTOR.

Inhibitors of mTOR were evaluated in AML. A caveat of mTOR inhibition is the resultant increased phosphorylation of AKT [50]. To circumvent this drawback, dual PI3K and mTOR inhibitors have been developed. Dactolisib, a dual PI3K/mTOR inhibitor, reduced cell growth and induced apoptosis in AML cells without affecting normal stem cell function [56]. The impact of combination therapy across the spectrum of PI3K/AKT/mTOR inhibitors is being elucidated. An overview of several PI3K pathway inhibitors is provided in Figure 3.

There is a relative paucity of data on outcomes of patients with AML harboring a PI3K pathway mutation treated with venetoclax-based strategies. The clinical impact of aberrations of PI3K is inferred from upstream effectors, such as FLT3 or KIT. These upstream kinase-activating mutations are associated with reduced survival when treated with venetoclax-based strategies, suggesting additive mechanisms of primary or adaptive resistance [7,50].

### Overcoming PI3K pathway resistance

Venetoclax resistance in AML with constitutively activated PI3K directly results from the upregulation of multiple anti-apoptotic mediators. In addition to AKT-dependent increases in BCL-2 and BCL-X_L_, a third anti-apoptotic protein, MCL-1, is also upregulated by the PI3K pathway [57]. Therefore, constitutive PI3K signaling promotes multiple avenues for venetoclax resistance through the upregulation of alternative anti-apoptotic mediators, facilitating cell survival despite BCL-2 inhibition.

To combat multiple resistance mechanisms mediated through PI3K, apitolisib, a dual PI3K/mTOR inhibitor, was studied in AML cells undergoing treatment with concurrent BCL-2 inhibition. Venetoclax and apitolisib induced rapid AML cell apoptosis with MCL-1 downregulation and spared normal hematopoietic cells [58]. The combination of venetoclax and apitolisib was also effective in venetoclax-resistant cells. These findings support the notion that the downregulation of MCL-1 is a consequence of targeting PI3K and BCL-2 rather than a result of cell death. The success of co- targeting PI3K and BCL-2 reinforced the results of additional studies that demonstrated resistant AML cell lines can be re-sensitized to venetoclax [29,59].

Activation of the PI3K pathway also plays a unique role in *FLT3*^mut^ AML undergoing treatment with FLT3 inhibition. *FLT3*-ITD AML cell lines resistant to sorafenib are enriched in the PI3K/mTOR pathway [60]. Gedatolisib blocked cell proliferation, induced apoptosis in resistant cell lines, and extended overall survival in PDX models following sorafenib exposure. These findings suggest that PI3K inhibition may be a subsequent therapeutic avenue to overcome resistance in patients initially treated with novel triplet regimens, such as a FLT3 inhibitor, venetoclax, and a hypomethylating agent. Further clinical trials should explore the efficacy of targeting the PI3K pathway to overcome or delay therapeutic resistance in patients treated with venetoclax-based strategies.

## RAS

### Overview of RAS

The RAS proteins exhibit substantial crosstalk with the PI3K pathway and stimulate cell proliferation and survival through the cascade of RAF, MEK, and ERK, which constitute the RAS pathway [53]. RAS pathway mutations commonly emerge as cooperating mutations in leukemogenesis — dance partners to additional mutations which accelerate subclonal evolution and promote resistance to therapy [61,62].

Similar to PI3K, constitutively activated RAS signaling frequently occurs in the context of upstream FLT3 activity, with downstream RAS activation contributing to FLT3 inhibitor resistance [63,64]. In addition, as RAS activity is guanine-5-triphosphate (GTP)-dependent, mutations that inactivate RAS GTPases will also result in constitutive RAS activation [65].

In AML, RAS mutations confer a robust proliferative advantage and skew differentiation toward the myelomonocytic line [66,67]. Patients with *RAS*^mut^ AML have a significantly longer overall survival than *RAS* wild-type, perhaps partly due to a higher likelihood of association with favorable-risk cytogenetics [68]. In particular, *RAS*^mut^ AML demonstrated significantly longer overall survival when treated with intensive cytarabine-containing induction regimens. Intensive chemotherapy with venetoclax did not significantly improve response rates in *RAS*^mut^ AML compared to intensive chemotherapy without venetoclax. In contrast, response rates, but not survival, were significantly lower in patients harboring a *RAS* mutation treated with a hypomethylating agent and venetoclax [68]. These findings support the hypothesis of RAS-mediated venetoclax resistance when treated in combination with a hypomethylating agent.

Clinical trials evaluated multiple inhibitors of the RAS pathway. Selumetinib, a MEK inhibitor, was associated with modest single-agent activity in relapsed or refractory AML, but no patient with *FLT3*-ITD responded [69]. Resistance to MEK inhibition in *FLT3*-ITD AML suggests that the activation of parallel signaling pathways contributes to disease refractoriness, warranting the investigation of combination strategies. Indeed, MEK inhibition with trametinib enhanced the response to FLT3 inhibitors, improving survival in mouse models while sparing normal CD34^+^ cells [70]. Therefore, the notion that RAS pathway-targeting combination strategies may improve survival while reducing treatment resistance is gaining momentum.

### Overcoming RAS resistance

AML with myelomonocytic differentiation is implicated in venetoclax resistance, raising the hypothesis that RAS activation may be a driving factor [71]. Similar to constitutive FLT3 signaling, the RAS pathway contributes to MCL-1 stabilization through post-translational modification, ultimately resulting in venetoclax resistance [11,72,73]. This discovery prompted the investigation of combination strategies evaluating the efficacy of venetoclax with inhibitors of RAS or MEK.

Further studies in AML cell lines evaluated the combination of venetoclax and cobimetinib. Using this strategy, MCL-1 was downregulated following MEK inhibition, which sensitized cells to venetoclax and culminated in reduced leukemia cell burden in mouse models [14]. Next, MEK inhibitors were evaluated in venetoclax-resistant cells. Upon exposure of venetoclax-resistant cells to cobimetinib, the level of MCL-1 decreased only minimally, suggesting activation of pathways upstream of MEK [11].

Subsequently, *RAS* mutations and MAPK activation were discovered to stabilize MCL-1, resulting in persistent cell survival. To confirm the role of MCL-1 in RAS-mediated resistance to venetoclax, co-treatment with venetoclax and an MCL-1 inhibitor, AZD-5991, significantly reduced AML blasts in mice [11]. These findings formed the foundation for clinical trials co-targeting BCL-2 and the RAS pathway in *RAS*^mut^ AML.

Based on this data, a clinical trial evaluated trametinib with venetoclax and 7-day azacitidine in relapsed or refractory *RAS*^mut^ AML. Sixteen patients received a median of four lines of prior therapy, and 81% received a prior hypomethylating agent with venetoclax. While 67% of patients that did not receive venetoclax-based treatment responded, only 15% of patients with previous venetoclax exposure had a response [15]. The median overall survival was 2.4 months, and high-grade adverse events occurred in half of those treated [15], resulting in premature study closure due to low efficacy and high toxicity.

Therefore, more specific and less toxic inhibitors of the RAS pathway are needed to improve survival in this cohort.

## Conclusion

Venetoclax revolutionized the treatment of AML and ushered in new waves of questions. The most glaring question is readily apparent: how can we overcome or delay venetoclax resistance? Resistance to venetoclax has been consistently observed across molecular cohorts, primarily through the upregulation of MCL-1 and BCL-X_L_. Signaling pathways commonly activated in AML frequently result in the elevation of these alternative anti-apoptotic mediators, culminating in treatment failure and disease relapse. These biological contributors of resistance are directly implicated in the observation of decreased overall survival in patients treated with venetoclax harboring constitutively activated signaling mutations [7].

Among the three commonly activated signaling pathways in AML — FLT3, PI3K, and RAS — the most promising therapeutic approach appears to be targeting FLT3 with a triplet regimen. The pre-clinical observation that FLT3 inhibitor-mediated MCL-1 downregulation is synergistic with BCL-2 inhibition illuminated the path to impressive early phase clinical trial results. Incorporating gilteritinib into triplet therapy with venetoclax offers a promising therapeutic advancement, particularly in the first-line setting. In this context, FLT3 inhibition may delay venetoclax resistance. In contrast, the optimal strategy at the time of disease relapse following exposure to a FLT3 inhibitor and venetoclax is unclear. Two therapeutic avenues can be envisioned: targeting the newly emerged mutation contributing to resistance or adopting a novel strategy. Innovative FLT3-directed therapies offer alternative methods of attack: FLT3 bispecific T-cell engagers (BiTEs) and FLT3-directed chimeric antigen receptor (CAR) T-cell therapy [74,75].

Specific, efficacious, and potent inhibition of PI3K and RAS has yet to be fully realized in AML, and the optimal combinations and targets remain to be explored. This leads to a second question: what are the optimal targets for these pathways, and how should they be built into existing treatment strategies for patients who are not candidates for intensive chemotherapy? While venetoclax-based triplet combinations are compelling, clinical trials have not yet supported this approach for AML with mutated *PI3K* or *RAS*. Sequencing targeted therapy after the emergence of activating *PI3K* or *RAS* mutations following venetoclax exposure may improve survival and reduce toxicity. Alternatively, novel approaches hold promise — such as RAS-directed CAR T-cell therapy [76].

The development of new therapies in AML has accelerated rapidly in recent years. Despite this, novel agents are still sorely needed and eagerly anticipated. Alternatively, direct inhibition of MCL-1 or other anti-apoptotic mediators may provide additional avenues for overcoming signaling pathway-mediated resistance. Regardless, it is an exciting era in the treatment of AML — one with many new therapeutic combination strategies waiting to be explored.

## Abbreviations:

AML: Acute Myeloid Leukemia
BCL-2: B-cell Lymphoma 2
IDH1: Isocitrate Dehydrogenase 1
IDH2: Isocitrate Dehydrogenase 2
FLT3: FMS-Like Tyrosine kinase 3
LSC: Leukemic Stem Cell
MAPK: Mitogen-Activated Protein Kinase
mTOR: Mammalian Target of Rapamycin
RAS: Rat Sarcoma
PI3K: Phosphatidylinositol-3-kinase

## References

[1] Bouligny IM, Maher KR, Grant S. Mechanisms of myeloid leukemogenesis: Current perspectives and therapeutic objectives. Blood Reviews. 2022 Aug 2:100996. 35989139 10.1016/j.blre.2022.100996 PMC10693933

[2] Oran B, Weisdorf DJ. Survival for older patients with acute myeloid leukemia: a population-based study. Haematologica. 2012 Dec;97(12):1916. 22773600 10.3324/haematol.2012.066100 PMC3590098

[3] Song X, Peng Y, Wang X, Chen Y, Jin L, Yang T, Incidence, survival, and risk factors for adults with acute myeloid leukemia not otherwise specified and acute myeloid leukemia with recurrent genetic abnormalities: analysis of the surveillance, epidemiology, and end results (SEER) database, 2001–2013. Acta Haematologica. 2018;139(2):115–27. 29455198 10.1159/000486228

[4] Cortes JE, Heidel FH, Hellmann A, Fiedler W, Smith BD, Robak T, Randomized comparison of low dose cytarabine with or without glasdegib in patients with newly diagnosed acute myeloid leukemia or high-risk myelodysplastic syndrome. Leukemia. 2019 Feb;33(2):379–89. 30555165 10.1038/s41375-018-0312-9 PMC6365492

[5] DiNardo CD, Jonas BA, Pullarkat V, Thirman MJ, Garcia JS, Wei AH, Azacitidine and venetoclax in previously untreated acute myeloid leukemia. New England Journal of Medicine. 2020 Aug 13;383(7):617–29. 32786187 10.1056/NEJMoa2012971

[6] Bouligny IM, Mehta V, Isom S, Ellis LR, Bhave RR, Howard DS, Lyerly S, Manuel M, Dralle S, Powell BL, Pardee TS. Efficacy of 10-day decitabine in acute myeloid leukemia. Leukemia Research. 2021 Apr 1;103:106524. 33640708 10.1016/j.leukres.2021.106524 PMC9006187

[7] DiNardo CD, Tiong IS, Quaglieri A, MacRaild S, Loghavi S, Brown FC, Molecular patterns of response and treatment failure after frontline venetoclax combinations in older patients with AML. Blood. 2020 Mar 12;135(11):791–803. 31932844 10.1182/blood.2019003988 PMC7068032

[8] Konopleva MY. Mechanisms for resistance in AML insights into molecular pathways mediating resistance to venetoclax. Best Practice & Research Clinical Haematology. 2021 Mar 1;34(1):101251. 33762105 10.1016/j.beha.2021.101251

[9] Salah HT, DiNardo CD, Konopleva M, Khoury JD. Potential biomarkers for treatment response to the Bcl-2 inhibitor venetoclax: State of the art and future directions. Cancers. 2021 Jun 14;13(12):2974. 34198580 10.3390/cancers13122974 PMC8231978

[10] Ong F, Kim K, Konopleva MY. Venetoclax resistance: mechanistic insights and future strategies. Cancer Drug Resistance. 2022;5(2):380–400. 35800373 10.20517/cdr.2021.125 PMC9255248

[11] Zhang Q, Riley-Gillis B, Han L, Jia Y, Lodi A, Zhang H, Activation of RAS/MAPK pathway confers MCL-1 mediated acquired resistance to BCL-2 inhibitor venetoclax in acute myeloid leukemia. Signal Transduction and Targeted Therapy. 2022 Feb 21;7(1):51. 35185150 10.1038/s41392-021-00870-3 PMC8858957

[12] Maiti A, DiNardo CD, Daver NG, Rausch CR, Ravandi F, Kadia TM, Triplet therapy with venetoclax, FLT3 inhibitor and decitabine for FLT3-mutated acute myeloid leukemia. Blood Cancer Journal. 2021 Feb 1;11(2):25. 33563904 10.1038/s41408-021-00410-w PMC7873265

[13] Short NJ, DiNardo CD, Daver N, Nguyen D, Yilmaz M, Kadia TM, A triplet combination of azacitidine, venetoclax and gilteritinib for patients with FLT3-mutated acute myeloid leukemia: results from a phase I/II study. Blood. 2021 Nov 23;138:696.

[14] Han L, Zhang Q, Dail M, Shi C, Cavazos A, Ruvolo VR, Concomitant targeting of BCL2 with venetoclax and MAPK signaling with cobimetinib in acute myeloid leukemia models. Haematologica. 2020 Mar;105(3):697–707. 31123034 10.3324/haematol.2018.205534 PMC7049339

[15] Desikan SP, Ravandi F, Pemmaraju N, Konopleva M, Loghavi S, Jabbour EJ, A Phase II Study of Azacitidine, Venetoclax, and Trametinib in Relapsed or Refractory Acute Myeloid Leukemia Harboring RAS Pathway-Activating Mutations. Acta Haematologica. 2022;145(5):529–36. 35717939 10.1159/000525566

[16] Kevlicius L, Cepulyte R, Vasilevska D, Griskevicius L, Zucenka A. Venetoclax-based regimens in combination with trametinib for RAS-mutated relapsed or refractory myeloid malignancies. Bone Marrow Transplantation. 2022 Apr 16:1034–7. 35430592 10.1038/s41409-022-01679-6

[17] Lagadinou ED, Sach A, Callahan K, Rossi RM, Neering SJ, Minhajuddin M, BCL-2 inhibition targets oxidative phosphorylation and selectively eradicates quiescent human leukemia stem cells. Cell Stem Cell. 2013 Mar 7;12(3):329–41. 23333149 10.1016/j.stem.2012.12.013 PMC3595363

[18] Kale J, Osterlund EJ, Andrews DW. BCL-2 family proteins: changing partners in the dance towards death. Cell Death & Differentiation. 2018 Jan;25(1):65–80. 29149100 10.1038/cdd.2017.186 PMC5729540

[19] Kist M, Vucic D. Cell death pathways: intricate connections and disease implications. The EMBO Journal. 2021 Mar 1;40(5):e106700. 33439509 10.15252/embj.2020106700 PMC7917554

[20] Stein JC, Hansen G. Mannose induces an endonuclease responsible for DNA laddering in plant cells. Plant Physiology. 1999 Sep;121(1):71–80. 10482662 10.1104/pp.121.1.71 PMC59391

[21] Li P, Nijhawan D, Budihardjo I, Srinivasula SM, Ahmad M, Alnemri ES, Cytochrome c and dATP-dependent formation of Apaf-1/caspase-9 complex initiates an apoptotic protease cascade. Cell. 1997 Nov 14;91(4):479–89. 9390557 10.1016/s0092-8674(00)80434-1

[22] Bose P, Gandhi V, Konopleva M. Pathways and mechanisms of venetoclax resistance. Leukemia & Lymphoma. 2017 Sep 2;58(9):2026–39. 10.1080/10428194.2017.1283032 PMC5478500 28140720

[23] Kuwana T, Bouchier-Hayes L, Chipuk JE, Bonzon C, Sullivan BA, Green DR, BH3 domains of BH3-only proteins differentially regulate Bax-mediated mitochondrial membrane permeabilization both directly and indirectly. Molecular Cell. 2005 Feb 18;17(4):525–35. 15721256 10.1016/j.molcel.2005.02.003

[24] Shamas-Din A, Kale J, Leber B, Andrews DW. Mechanisms of action of Bcl-2 family proteins. Cold Spring Harbor perspectives in biology. 2013 Apr 1;5(4):a008714. 23545417 10.1101/cshperspect.a008714 PMC3683897

[25] Guerra VA, DiNardo C, Konopleva M. Venetoclax-based therapies for acute myeloid leukemia. Best Practice & Research Clinical Haematology. 2019 Jun 1;32(2):145–53. 31203996 10.1016/j.beha.2019.05.008 PMC6581210

[26] Cheng EH, Wei MC, Weiler S, Flavell RA, Mak TW, Lindsten T, Korsmeyer SJ. BCL-2, BCL-X(L) sequester BH3 domain-only molecules preventing BAX- and BAK-mediated mitochondrial apoptosis. Molecular Cell. 2001 Sep;8(3):705–11. 11583631 10.1016/s1097-2765(01)00320-3

[27] Cory S, Adams JM. The Bcl2 family: regulators of the cellular life-or-death switch. Nature Reviews Cancer. 2002 Sep;2(9):647–56. 12209154 10.1038/nrc883

[28] Kaefer A, Yang J, Noertersheuser P, Mensing S, Humerickhouse R, Awni W, Mechanism-based pharmacokinetic/pharmacodynamic meta-analysis of navitoclax (ABT-263) induced thrombocytopenia. Cancer Chemotherapy and Pharmacology. 2014 Sep;74(3):593–602. 25053389 10.1007/s00280-014-2530-9

[29] Lin KH, Winter PS, Xie A, Roth C, Martz CA, Stein EM, Targeting MCL-1/BCL-XL forestalls the acquisition of resistance to ABT-199 in acute myeloid leukemia. Scientific Reports. 2016 Jun 10;6(1):27696. 27283158 10.1038/srep27696 PMC4901329

[30] ThijssenR, TianL, AndersonMA, FlensburgC, JarrattA, GarnhamAL, Single-cell multiomics reveal the scale of multilayered adaptations enabling CLL relapse during venetoclax therapy. Blood, The Journal of the American Society of Hematology. 2022 Nov 17;140(20):2127–41.10.1182/blood.202201604035709339

[31] Condoluci A, Rossi D. Mechanisms of resistance to venetoclax. Blood, The Journal of the American Society of Hematology. 2022 Nov 17;140(20):2094–6. 10.1182/blood.2022017341 36394906

[32] ThomallaD, BeckmannL, GrimmC, OliverioM, MederL, HerlingCD, Deregulation and epigenetic modification of BCL2-family genes cause resistance to venetoclax in hematologic malignancies. Blood, The Journal of the American Society of Hematology. 2022 Nov 17;140(20):2113–26.10.1182/blood.202101430435704690

[33] Grafone T, Palmisano M, Nicci C, Storti S. An overview on the role of FLT3-tyrosine kinase receptor in acute myeloid leukemia: biology and treatment. Oncology reviews. 2012 Mar 5;6(1):8. 10.4081/oncol.2012.e8 PMC4419636 25992210

[34] van der Geer P, Hunter T, Lindberg RA. Receptor protein-tyrosine kinases and their signal transduction pathways. Annual review of cell biology. 1994 Nov;10(1):251–337. 10.1146/annurev.cb.10.110194.001343 7888178

[35] Daver N, Schlenk RF, Russell NH, Levis MJ. Targeting FLT3 mutations in AML: review of current knowledge and evidence. Leukemia. 2019 Feb;33(2):299–312. 30651634 10.1038/s41375-018-0357-9 PMC6365380

[36] Kiyoi H, Naoe T, Nakano Y, Yokota S, Minami S, Miyawaki S, Prognostic implication of FLT3 and N-RAS gene mutations in acute myeloid leukemia. Blood, The Journal of the American Society of Hematology. 1999 May 1;93(9):3074–80. 10216104

[37] Kottaridis PD, Gale RE, Frew ME, Harrison G, Langabeer SE, Belton AA, The presence of a FLT3 internal tandem duplication in patients with acute myeloid leukemia (AML) adds important prognostic information to cytogenetic risk group and response to the first cycle of chemotherapy: analysis of 854 patients from the United Kingdom Medical Research Council AML 10 and 12 trials. Blood, The Journal of the American Society of Hematology. 2001 Sep 15;98(6):1752–9. 10.1182/blood.v98.6.1752 11535508

[38] Man CH, Fung TK, Ho C, Han HH, Chow HC, Ma AC, Sorafenib treatment of FLT3-ITD+ acute myeloid leukemia: favorable initial outcome and mechanisms of subsequent nonresponsiveness associated with the emergence of a D835 mutation. Blood, The Journal of the American Society of Hematology. 2012 May 31;119(22):5133–43. 10.1182/blood-2011-06-363960 22368270

[39] Röllig C, Serve H, Hüttmann A, Noppeney R, Müller-Tidow C, Krug U, Addition of sorafenib versus placebo to standard therapy in patients aged 60 years or younger with newly diagnosed acute myeloid leukaemia (SORAML): a multicentre, phase 2, randomised controlled trial. The Lancet Oncology. 2015 Dec 1;16(16):1691–9. 26549589 10.1016/S1470-2045(15)00362-9

[40] Stone RM, Mandrekar SJ, Sanford BL, Laumann K, Geyer S, Bloomfield CD, Midostaurin plus chemotherapy for acute myeloid leukemia with a FLT3 mutation. New England Journal of Medicine. 2017 Aug 3;377(5):454–64. 28644114 10.1056/NEJMoa1614359 PMC5754190

[41] Wander SA, Levis MJ, Fathi AT. The evolving role of FLT3 inhibitors in acute myeloid leukemia: quizartinib and beyond. Therapeutic Advances in Hematology. 2014 Jun;5(3):65–77. 24883179 10.1177/2040620714532123 PMC4031904

[42] Perl AE, Martinelli G, Cortes JE, Neubauer A, Berman E, Paolini S, Gilteritinib or chemotherapy for relapsed or refractory FLT3-mutated AML. New England Journal of Medicine. 2019 Oct 30. 10.1056/NEJMoa1902688 31665578

[43] Konopleva M, Thirman MJ, Pratz KW, Garcia JS, Recher C, Pullarkat V, Impact of FLT3 mutation on outcomes after venetoclax and azacitidine for patients with treatment-naive acute myeloid leukemia. Clinical Cancer Research. 2022 Jan 1. 10.1158/1078-0432.CCR-21-3405 PMC9365380 35063965

[44] Chyla B, Daver N, Doyle K, McKeegan E, Huang X, Ruvolo V, Genetic biomarkers of sensitivity and resistance to venetoclax monotherapy in patients with relapsed acute myeloid leukemia. American Journal of Hematology. 2018 Aug;93(8):E202. 29770480 10.1002/ajh.25146 PMC6120451

[45] Kasper S, Breitenbuecher F, Heidel F, Hoffarth S, Markova B, Schuler M, Targeting MCL-1 sensitizes FLT3-ITD-positive leukemias to cytotoxic therapies. Blood Cancer Journal. 2012 Mar;2(3):e60-. 22829255 10.1038/bcj.2012.5 PMC3317524

[46] Mali RS, Zhang Q, DeFilippis RA, Cavazos A, Kuruvilla VM, Raman J, Venetoclax combines synergistically with FLT3 inhibition to effectively target leukemic cells in FLT3-ITD+ acute myeloid leukemia models. Haematologica. 2021 Apr 1;106(4):1034. 32414851 10.3324/haematol.2019.244020 PMC8017817

[47] Yoshimoto G, Miyamoto T, Jabbarzadeh-Tabrizi S, Iino T, Rocnik JL, Kikushige Y, FLT3-ITD up-regulates MCL-1 to promote survival of stem cells in acute myeloid leukemia via FLT3-ITD–specific STAT5 activation. Blood, The Journal of the American Society of Hematology. 2009 Dec 3;114(24):5034–43. 10.1182/blood-2008-12-196055 PMC2788977 19808698

[48] Ma J, Zhao S, Qiao X, Knight T, Edwards H, Polin L, Inhibition of Bcl-2 Synergistically Enhances the Antileukemic Activity of Midostaurin and Gilteritinib in Preclinical Models of FLT3-Mutated Acute Myeloid LeukemiaJoint FLT3 and BCL-2 Inhibition in FLT3-Mutated AML. Clinical Cancer Research. 2019 Nov 15;25(22):6815–26. 31320594 10.1158/1078-0432.CCR-19-0832 PMC6858954

[49] Zhu R, Li L, Nguyen B, Seo J, Wu M, Seale T, FLT3 tyrosine kinase inhibitors synergize with BCL-2 inhibition to eliminate FLT3/ITD acute leukemia cells through BIM activation. Signal transduction and targeted therapy. 2021 May 24;6(1):186. 34024909 10.1038/s41392-021-00578-4 PMC8141515

[50] Darici S, Alkhaldi H, Horne G, Jørgensen HG, Marmiroli S, Huang X. Targeting PI3K/Akt/mTOR in AML: rationale and clinical evidence. Journal of Clinical Medicine. 2020 Sep 11;9(9):2934. 32932888 10.3390/jcm9092934 PMC7563273

[51] Yang J, Nie J, Ma X, Wei Y, Peng Y, Wei X. Targeting PI3K in cancer: mechanisms and advances in clinical trials. Molecular cancer. 2019 Dec;18(1):26. 30782187 10.1186/s12943-019-0954-x PMC6379961

[52] Karnauskas R, Niu Q, Talapatra S, Plas DR, Greene ME, Crispino JD, Bcl-xL and Akt cooperate to promote leukemogenesis in vivo. Oncogene. 2003 Feb;22(5):688–98. 12569361 10.1038/sj.onc.1206159

[53] Steelman LS, Pohnert SC, Shelton JG, Franklin RA, Bertrand FE, McCubrey JA. JAK/STAT, Raf/MEK/ERK, PI3K/Akt and BCR-ABL in cell cycle progression and leukemogenesis. Leukemia. 2004 Feb;18(2):189–218. 14737178 10.1038/sj.leu.2403241

[54] Vargaftig J, Farhat H, Ades L, Briaux A, Benoist C, Turbiez I, Phase 2 Trial of Single Agent Gedatolisib (PF-05212384), a Dual PI3K/mTOR Inhibitor, for Adverse Prognosis and Relapse/Refractory AML: Clinical and Transcriptomic Results. Blood. 2018 Nov 29;132:5233.

[55] Sampath D, Malik A, Plunkett W, Nowak B, Williams B, Burton M, Phase I clinical, pharmacokinetic, and pharmacodynamic study of the Akt-inhibitor triciribine phosphate monohydrate in patients with advanced hematologic malignancies. Leukemia Research. 2013 Nov 1;37(11):1461–7. 23993427 10.1016/j.leukres.2013.07.034 PMC4205589

[56] Chapuis N, Tamburini J, Green AS, Vignon C, Bardet V, Neyret A, Dual Inhibition of PI3K and mTORC1/2 Signaling by NVP-BEZ235 as a New Therapeutic Strategy for Acute Myeloid LeukemiaAntileukemic Activity of the NVP-BEZ235 Compound in AML. Clinical Cancer Research. 2010 Nov 15;16(22):5424–35. 20884625 10.1158/1078-0432.CCR-10-1102

[57] Wang JM, Chao JR, Chen W, Kuo ML, Yen JJ, Yang-Yen HF. The antiapoptotic gene mcl-1 is up-regulated by the phosphatidylinositol 3-kinase/Akt signaling pathway through a transcription factor complex containing CREB. Molecular and Cellular Biology. 1999 Sep 1;19(9):6195–206. 10454566 10.1128/mcb.19.9.6195 PMC84561

[58] Rahmani M, Nkwocha J, Hawkins E, Pei X, Parker RE, Kmieciak M. Cotargeting BCL-2 and PI3K Induces BAX-Dependent Mitochondrial Apoptosis in AML CellsVenetoclax/GDC-0980 BAX-Dependent Anti-AML Activity. Cancer Research. 2018 Jun 1;78(11):3075–86. 29559471 10.1158/0008-5472.CAN-17-3024 PMC5984704

[59] Choudhary GS, Al-Harbi S, Mazumder S, Hill BT, Smith MR, Bodo J, MCL-1 and BCL-xL-dependent resistance to the BCL-2 inhibitor ABT-199 can be overcome by preventing PI3K/AKT/mTOR activation in lymphoid malignancies. Cell Death & Disease. 2015 Jan;6(1):e1593-. 25590803 10.1038/cddis.2014.525 PMC4669737

[60] Lindblad O, Cordero E, Puissant A, Macaulay L, Ramos A, Kabir NN, Aberrant activation of the PI3K/mTOR pathway promotes resistance to sorafenib in AML. Oncogene. 2016 Sep;35(39):5119–31. 26999641 10.1038/onc.2016.41 PMC5399143

[61] Cancer Genome Atlas Research Network. Genomic and epigenomic landscapes of adult de novo acute myeloid leukemia. New England Journal of Medicine. 2013 May 30;368(22):2059–74. 10.1056/NEJMoa1301689 PMC3767041 23634996

[62] Carter JL, Hege K, Yang J, Kalpage HA, Su Y, Edwards H, Targeting multiple signaling pathways: the new approach to acute myeloid leukemia therapy. Signal Transduction and Targeted Therapy. 2020 Dec 18;5(1):1–29. 33335095 10.1038/s41392-020-00361-x PMC7746731

[63] Ward AF, Braun BS, Shannon KM. Targeting oncogenic Ras signaling in hematologic malignancies. Blood, The Journal of the American Society of Hematology. 2012 Oct 25;120(17):3397–406. 10.1182/blood-2012-05-378596 PMC3482854 22898602

[64] McMahon CM, Ferng T, Canaani J, Wang ES, Morrissette JJ, Eastburn DJ, Clonal Selection with RAS Pathway Activation Mediates Secondary Clinical Resistance to Selective FLT3 Inhibition in Acute Myeloid LeukemiaSecondary Resistance to Selective FLT3 Inhibition in AML. Cancer discovery. 2019 Aug 1;9(8):1050–63. 31088841 10.1158/2159-8290.CD-18-1453 PMC11994087

[65] Bos JL. Ras oncogenes in human cancer: a review. Cancer Research. 1989 Sep 1;49(17):4682–9. 2547513

[66] Van Meter ME, Díaz-Flores E, Archard JA, Passegué E, Irish JM, Kotecha N, K-RasG12D expression induces hyperproliferation and aberrant signaling in primary hematopoietic stem/progenitor cells. Blood. 2007 May 1;109(9):3945–52. 17192389 10.1182/blood-2006-09-047530 PMC1874575

[67] Fatrai S, Van Gosliga D, Han L, Daenen SM, Vellenga E, Schuringa JJ. KRASG12V enhances proliferation and initiates myelomonocytic differentiation in human stem/progenitor cells via intrinsic and extrinsic pathways. Journal of Biological Chemistry. 2011 Feb 25;286(8):6061–70. 21169357 10.1074/jbc.M110.201848 PMC3057777

[68] Rivera D, Kim K, Kanagal-Shamanna R, Borthakur G, Montalban-Bravo G, Daver N, Implications of RAS mutational status in subsets of patients with newly diagnosed acute myeloid leukemia across therapy subtypes. American Journal of Hematology. 2022 Dec;97(12):1599–606. 36117258 10.1002/ajh.26731 PMC11975417

[69] Jain N, Curran E, Iyengar NM, Diaz-Flores E, Kunnavakkam R, Popplewell L, Phase II study of the oral MEK inhibitor selumetinib (AZD6244) in advanced acute myeloid leukemia (AML). Clin Cancer Res, 2014. 20(2): p. 490–8. 24178622 10.1158/1078-0432.CCR-13-1311 PMC4310865

[70] Morales ML, Arenas A, Ortiz-Ruiz A, Leivas A, Rapado I, Rodríguez-García A, MEK inhibition enhances the response to tyrosine kinase inhibitors in acute myeloid leukemia. Scientific Reports. 2019 Dec 9;9(1):18630. 31819100 10.1038/s41598-019-54901-9 PMC6901485

[71] Zhang H, Nakauchi Y, Köhnke T, Stafford M, Bottomly D, Thomas R, Integrated analysis of patient samples identifies biomarkers for venetoclax efficacy and combination strategies in acute myeloid leukemia. Nature cancer. 2020 Aug;1(8):826–39. 33123685 10.1038/s43018-020-0103-x PMC7591155

[72] Konopleva M, Milella M, Ruvolo P, Watts JC, Ricciardi MR, Korchin B, MEK inhibition enhances ABT-737-induced leukemia cell apoptosis via prevention of ERK-activated MCL-1 induction and modulation of MCL-1/BIM complex. Leukemia. 2012 Apr;26(4):778–87. 22064351 10.1038/leu.2011.287 PMC3604791

[73] Zhang W, Ruvolo VR, Gao C, Zhou L, Bornmann W, Tsao T, Evaluation of Apoptosis Induction by Concomitant Inhibition of MEK, mTOR, and Bcl-2 in Human Acute Myelogenous Leukemia CellsABT-737 Enhances MEK/mTOR Inhibition-Induced AML Apoptosis. Molecular cancer therapeutics. 2014 Jul 1;13(7):1848–59. 24739393 10.1158/1535-7163.MCT-13-0576 PMC4090272

[74] Jetani H, Garcia-Cadenas I, Nerreter T, Thomas S, Rydzek J, Meijide JB, CAR T-cells targeting FLT3 have potent activity against FLT3–ITD+ AML and act synergistically with the FLT3-inhibitor crenolanib. Leukemia. 2018 May;32(5):1168–79. 29472720 10.1038/s41375-018-0009-0

[75] Brauchle B, Goldstein RL, Karbowski CM, Henn A, Li CM, Bücklein VL, Characterization of a Novel FLT3 BiTE Molecule for the Treatment of Acute Myeloid LeukemiaNovel FLT3 BiTE Molecule for AML Treatment. Molecular Cancer Therapeutics. 2020 Sep 1;19(9):1875–88. 32518207 10.1158/1535-7163.MCT-19-1093

[76] Levin N, Paria BC, Vale NR, Yossef R, Lowery FJ, Parkhurst MR, Identification and validation of T-cell receptors targeting RAS hotspot mutations in human cancers for use in cell-based immunotherapy. Clinical Cancer Research. 2021 Sep 15;27(18):5084–95. 34168045 10.1158/1078-0432.CCR-21-0849 PMC8448939

